# Cell-free DNA as diagnostic and prognostic biomarkers for adult sepsis: a systematic review and meta-analysis

**DOI:** 10.1038/s41598-023-46663-2

**Published:** 2023-11-10

**Authors:** Awirut Charoensappakit, Kritsanawan Sae-khow, Pongpera Rattanaliam, Nuntanuj Vutthikraivit, Monvasi Pecheenbuvan, Suwasin Udomkarnjananun, Asada leelahavanichkul

**Affiliations:** 1 Medical Microbiology, Interdisciplinary and International Program, Graduate School, Chulalongkorn University, Bangkok, 10330 Thailand; 2 Center of Excellence on Translational Research in Inflammation and Immunology (CETRII), Faculty of Medicines, Chulalongkorn University, Bangkok, 10330 Thailand; 3 Department of Clinical Microscopy, Faculty of Allied Health Sciences, Chulalongkorn University, Bangkok, 10330 Thailand; 4 Division of Critical Care Medicine, Department of Internal Medicine, Chulalongkorn University, Bangkok, 10330 Thailand; 5 Division of Nephrology, Department of Medicine, Faculty of Medicine, Chulalongkorn University, Bangkok, 10330 Thailand

**Keywords:** Diagnostic markers, Prognostic markers

## Abstract

Although cell-free DNA (cfDNA) is an emerging sepsis biomarker, the use of cfDNA, especially as diagnostic and prognostic indicators, has surprisingly not been systemically analyzed. Data of adult patients with sepsis that conducted cfDNA measurement within 24 h of the admission was collected from PubMed, ScienceDirect, Scopus, and Cochrane Library until October 2022. The Quality in Prognosis Studies (QUIPS) and Quality Assessment in Diagnostic Studies-2 (QUADAS-2) tools were used to reduce the risk of biased assessment. The mean difference (MD) of cfDNA concentration and the standardized mean difference (SMD) between populations was calculated using Review Manager (RevMan) version 5.4.1 package software. Pooled analysis from 18 included studies demonstrated increased serum cfDNA levels in sepsis when compared with healthy control (SMD = 1.02; 95% confidence interval (CI) 0.46–1.57) or non-sepsis patients in the intensive care unit (ICU) (SMD = 1.03; 95% CI 0.65–1.40), respectively. Meanwhile, a slight decrease in the statistical value was observed when compared with non-sepsis ICU patients with SIRS (SMD = 0.74; 95% 0.41–1.06). The lower cfDNA levels were also observed in sepsis survivors compared to the non-survivors (SMD at 1.43; 95%CI 0.69–2.17) with the pooled area under the receiver operating characteristic curve (AUC) of 0.76 (95% CI 0.64–0.87) for the mortality prediction. Levels of cfDNA showed a pooled sensitivity of 0.81 (95% CI 0.75–0.86) and specificity of 0.72 (95% CI 0.65–0.78) with pooled diagnostic odd ratio (DOR) at 25.03 (95% CI 5.48–114.43) for the identification of sepsis in critically ill conditions. The cfDNA levels were significantly higher in patients with sepsis and being a helpful indicator for the critically ill conditions of sepsis. Nevertheless, results of the test must be interpreted carefully with the context of all clinical situations.

## Introduction

Sepsis, a potentially fatal organ failure driven by a systemic infection, is a serious global health concern^1,2^. Immune reactions against pathogen-associated molecular patterns (PAMPs) from organisms or damage-associated molecular patterns (DAMPs) from injured host cells lead to abnormally uncontrolled inflammation during sepsis, which results in multiple organ failure and death^2,3^. Among several immune activations, cell-free DNA (cfDNA) or circulating free DNA, defined as the degraded 50–200 base pair (bp) DNA fragments released to the blood circulation, is initially described by Mandel et al*.* in 1948^4^. Then, serum or plasma level of cfDNA is demonstrated to have prognostic utility in several clinical conditions, especially in cancers^4,5^ and organ transplantations^6^, as a potential use for the non-invasive biomarker^7^. In several conditions, the cfDNA is originated from the damaged host cells, which can be subcategorized into nuclear DNA (nDNA) and mitochondrial DNA (mtDNA), that might have some useful characteristics as a biomarker. In cancer, cfDNA has been proposed to be a noninvasive biopsy representing the disease burdens and the overall survival predictors as the higher cfDNA might be due to higher burdens of the malignant cells^7,8^. In prenatal diagnosis, cfDNA is used to screen chromosome abnormalities of the fetus as detection of fetal cfDNA in the mother's blood is a non-invasive procedure for fetal anomalies^9^.

Because cfDNA is possibly associated with cell injury, cfDNA might be a biomarker of cell damage in several conditions, including i) non-infectious inflammation (trauma, myocardial infarction, stroke, transplantation, diabetes, and sickle cell disease) and ii) infectious causes (multi-organ injury in sepsis)^10^. Moreover, small DNA fragments produced by the spontaneous breakdown of the cfDNA can activate pattern recognition receptors (PRRs) on both the cell surface and in the endosome. This cfDNA in sepsis induce more prominent innate immune responses through several PRRs, including Toll-like receptors (TLRs) in the endosomes (TLR-3, TLR-7, TLR-8, and TLR-9) and on the cell surface (TLR-4) and the intra-cytosolic receptors, including cyclic GMP–AMP synthase (cGAS)-stimulator of interferon genes (STING), retinoic acid-inducible gene I (RIG-1), and melanoma differentiation-associated protein 5 (MDA5)^11^. Indeed, cfDNA is proposed as a biomarker to distinguish sepsis and to predict sepsis mortality may be significant in sepsis.

Because an early identification of severe sepsis might lead to prompt interventions that may reduce sepsis mortality^12–14^, the prognostic and diagnostic biomarkers in sepsis are clinically useful. Although blood culture is a gold standard for diagnosis of severe infection, only 30% of blood culture in patients with sepsis is positive^15,16^. Then, other additional biomarkers are needed. In the intensive care unit (ICU), cfDNA has received increasing attention in sepsis, as the prognostic and predictive biomarkers, under a hypothesis that cfDNA originates from the dead cells and immune cells during sepsis and abundance of the dead cells might be correlated with sepsis severity^7,17^. Currently, the use of cfDNA as a sepsis biomarker has only been reported in several clinical studies without a systemic analysis, while physicians require a comprehensive evaluation of cfDNA in sepsis. Thus, the aim of this study was to evaluate the potential of cfDNA as a diagnostic biomarker to identify sepsis from other critical illness and prognostic capacity to predict sepsis mortality in patients with sepsis using systemic review and meta-analysis.

## Methods

This study was conducted following the Preferred Reporting Items for Systematic Reviews and Meta-analyses (PRISMA) 2020 guidelines.

### Search strategy

A systematic literature search of the Scopus, ScienceDirect, PubMed, and Cochrane Library databases published from 2000 onwards until October 2022 was performed. The article was limited only to the studies conducted on humans and reported in the English language. The retrieving query formulation used for the search were “cell free DNA” OR “cfDNA” AND “sepsis” AND “human”. All the reference lists of the identified articles and relevant reviews were also manually screened. Two groups of reviewers independently screened the inclusion of the article’s eligibility. The discordance in any topics during the review process was resolved through a consensus with the third group of reviewers.

### Eligibility criteria

The inclusion criteria were cohort human studies investigating diagnostic or prognostic accuracy of cfDNA in plasma or serum in sepsis of adults (aged ≥ 18 years old). Sepsis was defined as the standardized criteria, including Sepsis-1, Sepsis-2, or Sepsis-3, depending on the periods of publication^12^. Sepsis meant “life-threatening organ dysfunction caused by a dysregulated host response to infection,” according to the Sepsis-3 definition and severe sepsis form the Sepsis-1 and Sepsis-2. Positive blood culture alone was also identified as sepsis because of the requirement for urgent antimicrobial therapy in these patients to prevent a life-threatening complication. A quality determination of the DNA samples and the processes of cfDNA detections, including quantified polymerase chain reaction (qPCR), fluorescent-based method, or spectroscopy, were thoroughly assessed in all enrolled studies. The exclusion criteria were case reports, reviews, conference abstracts, and preprint articles. The potentially eligible articles were reviewed independently by 2 groups of reviewers to confirm their eligibility. Citation screening and selections were documented and summarized in a PRISMA-compliant flow chart.

### Data extraction and analysis

The following data were extracted from the published articles as following: (i) general study information: author, year, country, study design, clinical setting; (ii) patient characteristics: sample size, age, gender proportion, sepsis definition, sepsis severity; (iii) biomarker measurement: time point of measurement and test method; (iv) mortality: follow-up duration and rate of mortality; (v) outcome measurement: biomarker concentration, the area under the receiver operating characteristic curve (AUC) for diagnosis and prediction of mortality with cut-off point together with sensitivity, and specificity. The data were recorded independently by two groups of authors on separate copies of the records, any discrepancies were determined by a consensus with the third group of reviewers. The Quality assessment was conducted using the Quality in Prognosis Studies (QUIPS) tool for prognostic studies^18^, while the Quality Assessment of Diagnostic Accuracy Studies 2 (QUADAS-2) tool for diagnostic studies^19^.

For pooling of the results, mean with standard deviations (SD) values were used for the calculations and the standard errors (SE) were calculated into SD using the Cochrane Collaboration formula (SD = SE x $\surd N$). For the values presented with median and range or interquartile range (IQR), the mean values and SD were estimated by statistical formula from Wan et al.^20^. Then, the results of the analysis were presented as the forest plots of pooled mean differences (MD) with 95%confidence interval. Statistical significance was defined at the *p*-value < 0.05. Heterogeneity was measured using the among-study variance (τ^2^), χ^2^ test, and I^2^ statistical analyses. For the measurement with an I^2^ < 50%, the results were pooled using a fixed effects model, otherwise a random effects model was used. All statistical analyses were performed using Review Manager (Revman) 5.4.1 package software.

## Results

### Study selection and characteristics

**Figure 1:**
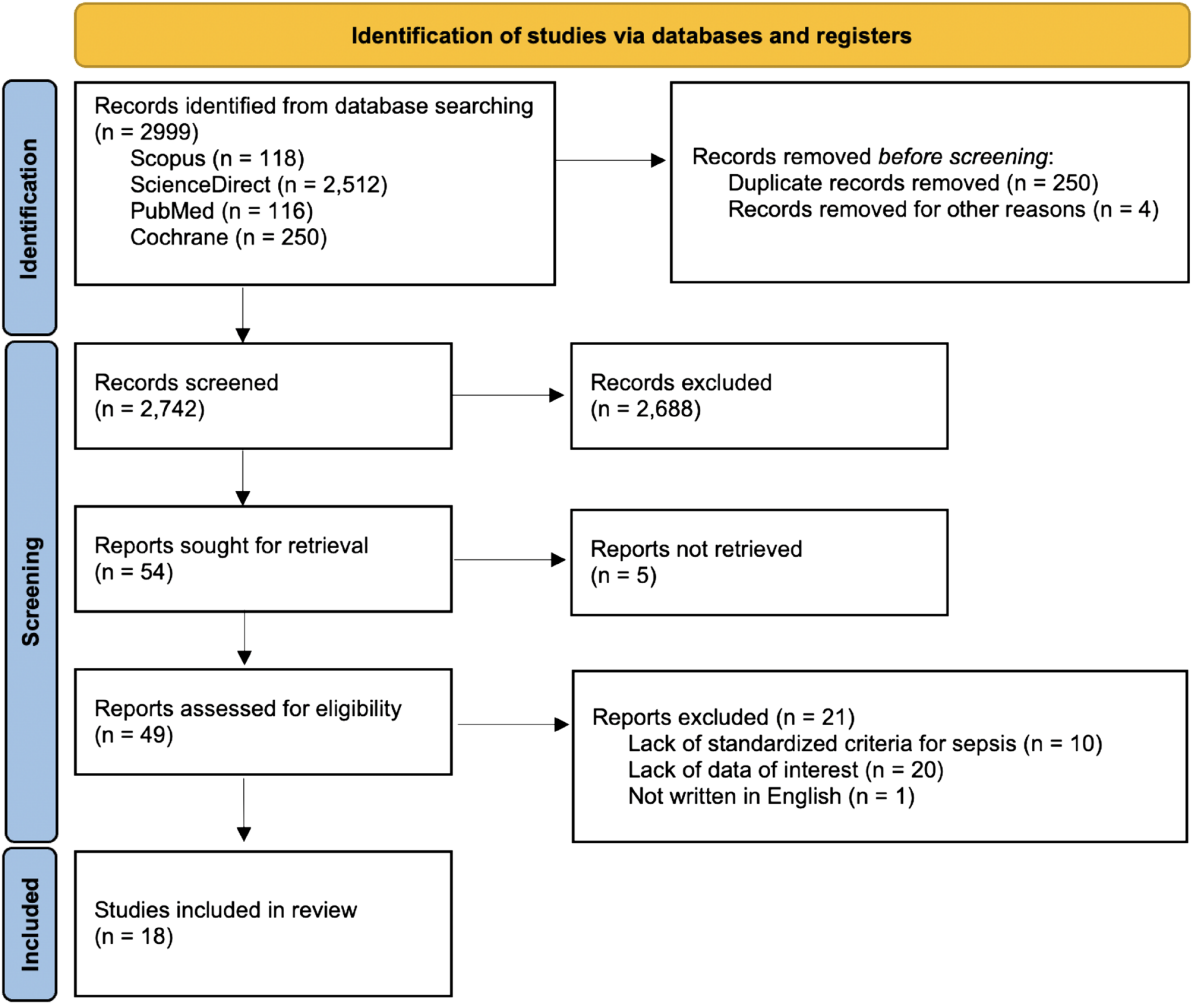
Preferred Reporting Items for Systematic Review and Meta-analyses (PRISMA) 2020 flow chart.

The systematic literature search retrieved 2742 articles. After initial screening by the title and abstract, 2688 articles were excluded. The full texts of the remaining 49 articles were examined and 21 studies were further excluded. Hence, 18 studies (17 prospective studies and 1 retrospective observation) with cfDNA analysis in adult blood samples at admission or at enrollment (published between 2006 and 2022) were included in the current meta-analysis (Fig. 1). There were nine and six studies from Europe and Eastern Asia, respectively, with two studies from North America and one study from Middle East Asia. The cfDNA detection methods, include ELISA (2 studies), spectroscopy (2 studies), fluorescent-based assay (6 studies), and qPCR (8 studies). The main characteristics of these studies are summarized in Table 1. A total of 1850 participants (healthy volunteers, patients with non-sepsis, and patients with sepsis) were enrolled with the number of participants across studies ranging from 3 to 221 cases. There were eight and four studies that examined the prognosis and diagnostic outcomes, respectively, and the remaining four studies simultaneously analyzed for both prognosis and diagnostic outcomes (Table 1).

**Table 1:** Characteristics of included studies.

Author	Year	Country	Study decide	Setting	Sepsis definition	Time to measurement	cfDNA protocol	Number of participants			Diagnostic or prognostic	Diagnostic outcome	Prognostic outcome
								Healthy	Non-sepsis ICU	Sepsis			
Andrew Rhodes et al*.*^21^	2006	UK	Prospective	ICU	Sepsis-1	ICU admission	qPCR	10	33	19	Prognostic	NA	ICU mortality
Anna Clementi et al*.*^22^	2016	Italy	Prospective	ICU	Sepsis-2	ICU admission	qPCR	NA	7	27	Prognostic	NA	28-day mortality
Avital Avirel et al*.*^23^	2014	Israel	Prospective	ICU	Sepsis-2	within 12 h after sepsis	Fluorescent	NA	NA	108	Prognostic	NA	hospital mortality
Basak Ceyda MECO et al*.*^24^	2013	Turkey	Prospective	ICU	Sepsis-1	ICU admission	qPCR	11	31 (SIRS)	11	Diagnostic	Sepsis vs. SIRS	NA
Chia-Te Kung et al*.*^25^	2012	Taiwan	Prospective	ICU, ED	Sepsis-1	within 24 h of admission	qPCR	33	NA	67	Prognostic	NA	Hospital mortality
Chistopher Duplessis et al*.*^26^	2018	US	Prospective	ICU, ED	Sepsis-2	within 24 h after sepsis	Fluorescent	NA	24 (SIRS)	131	Both	Sepsis vs. SIRS	28-day mortality
Dhruva J Dwivedi et al*.*^27^	2012	Canada	Retrospective	ICU	Sepsis-1	within 24 h after sepsis	Spectroscopy	14	NA	80	Prognostic	NA	28-day mortality
Dun Ling Xia et al*.*^28^	2016	China	Prospective	EICU	ESICM	ICU admission	qPCR	30	NA	140	Prognostic	NA	28-day mortality
Emmanuel Schneck et al*.*^29^	2016	Germany	Prospective	ICU	Sepsis-2	within 6 h of ICU admission	Spectroscopy	10	10	15	NA	NA	NA
Jesus Beltran-Garcia et al.^30^	2021	Spain	Prospective	ICU	Bacteremia	within 24 h after sepsis	ELISA	17	9	27	Diagnostic	Sepsis vs. non-sepsis ICU	NA
Jose Garnacho-Montero et al*.*^31^	2014	Spain	Prospective	ICU	Sepsis-1	within 24 h after sepsis	qPCR	10	43 (SIRS)	147	Both	Sepsis vs. SIRS	ICU mortality
Katri Seukkonen et al*.*^32^	2008	Finland	Prospective	ICU	Sepsis-1	within 24 h after sepsis	qPCR	NA	NA	255	Prognostic	NA	ICU mortality
Qiuyu Jing et al*.*^33^	2022	China	Prospective	ICU	Sepsis-3	within 24 h of ICU admission	Fluorescent	3	15	30	Both	Sepsis vs. non-sepsis ICU	28-day mortality
QiXing Chen et al*.*^34^	2012	China	Prospective	ICU	Sepsis-2	within 24 h after sepsis	ELISA	NA	29	45	Diagnostic	Sepsis vs. non-sepsis ICU	NA
Sebastien Tanaka et al*.*^35^	2019	France	Prospective	ICU	Sepsis-2	NA	Fluorescent	NA	20	20	NA	NA	NA
Vanessa Garc ´ıa Moreira et al*.*^36^	2010	Spain	Prospective	ICU, ED	Sepsis-1	ICU or ED admossion	qPCR	NA	30	54	Both	Sepsis vs. non-sepsis ICU	Global mortality
Yan-Qiang Hou et al*.*^37^	2016	China	Prospective	ICU	Sepsis-1	Within 6 h after sepsis	Fluorescent	73	43 (SIRS)	24	Diagnostic	Sepsis vs. SIRS	NA
Yuki Maruchi et al*.*^38^	2018	Japan	Prospective	ICU	Sepsis-2	Within 24 h after sepsis	Fluorescent	13	NA	55	Prognostic	NA	28-day mortality

*UK* United Kingdom, *US* United State, *ICU* intensive care unit, *ED* emergency department, *EICU* emergency intensive care unit, *ESICM* European Society of Intensive Care Medicine, *qPCR* quantitative real-time polymerase chain reaction, *ELISA* enzyme-linked immunosorbent assay, *SIRS* systemic inflammatory response syndrome, *NA* not applicable.

### Quality of enrolled studies

**Figure 2:**
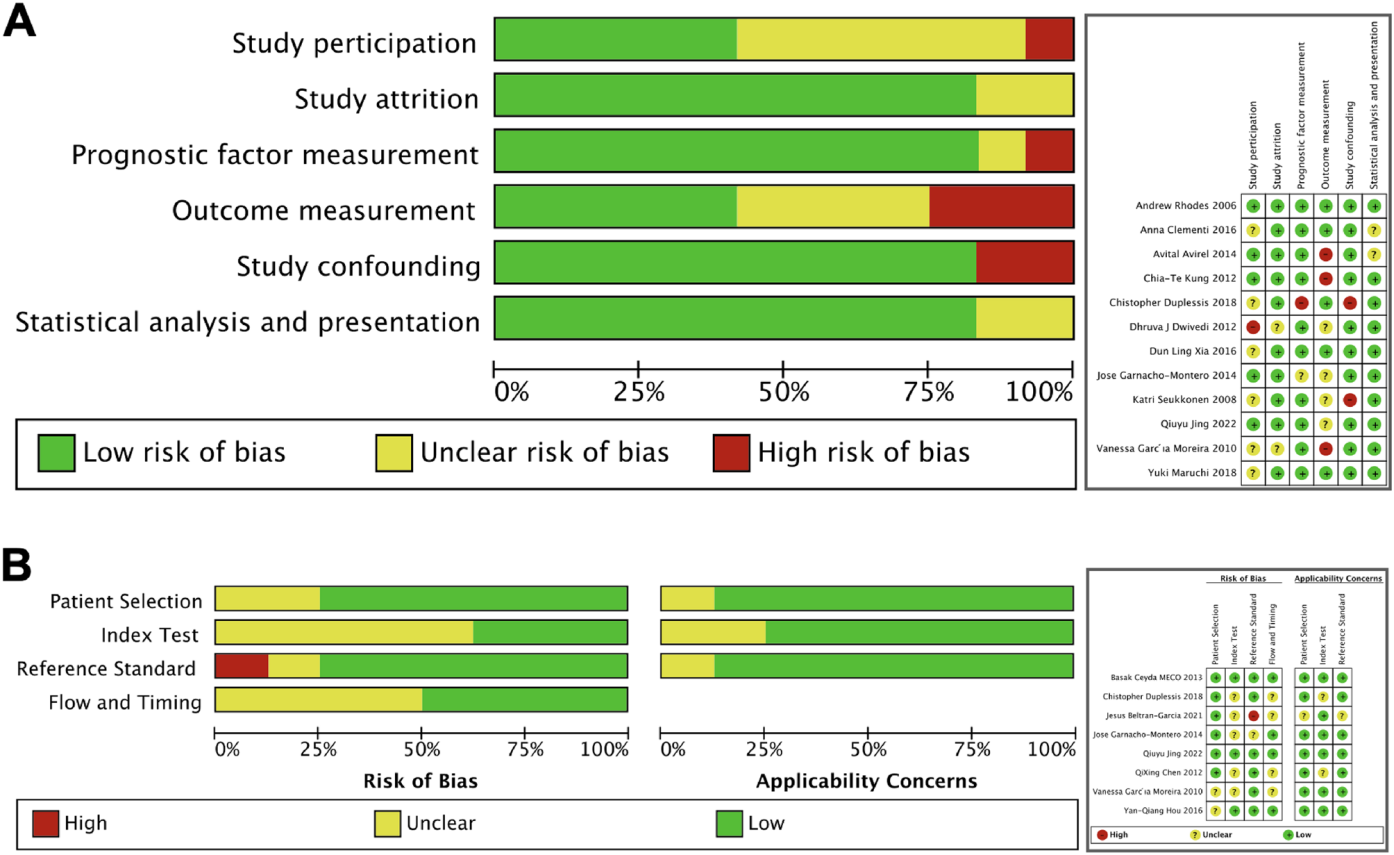
Quality assessment of included diagnostic studies, according to the QUIPS tool for prognostic studies and the QUADAS-2 tool for diagnostic studies.

The QUIPS and QUADAS-2 were used to evaluate prognostic and diagnostic studies, respectively^18,19^, as illustrated in Fig. 2. In the QUIPS measurement, 12 included prognostic studies^21–23,25–28,31–33,36,38^ with sepsis-related mortality were evaluated. Study participation and outcome measurement bias was identified as a concern in > 50% of the included studies. Many studies failed to specify whether they followed consecutive or random enrollment and reported no exclusion criteria. For outcome measurement, the risk of bias occurred due to the failure to clarify the time of measurement for the outcomes. There was a high-risk concern in three studies due to records of the hospital or global mortality which possibly did not relate to sepsis (Fig. 2A).

Eight studies with diagnostic outcomes^24,26,30,31,33,34,36,37^ were evaluated using QUADAS-2 measurement. For the index test domain of risk of bias, greater than 50% of studies were scored as unclear as these studies reported mean biomarker levels rather than AUC data, but cut-offs were not calculated. As well, it is also unclear if the index test was interpreted while blinded to the patient outcome. One study assigned a high risk for reference standard due to critically ill patients being included regardless of bacteremia, but all of the patients were not positive for blood culture. Four studies demonstrated an unclear risk of flow and timing bias due to the inappropriate timing of sample collection. Studies stated that biomarker was taken upon admission of study enrollment, but this specific time interval may vary for each patient and affect cfDNA levels studies. In this study, none of the studies had high concerns for applicability with respect to the reference standard (Fig. 2B).

### The difference in cfDNA concentration between sepsis, non-sepsis and healthy

**Figure 3:**
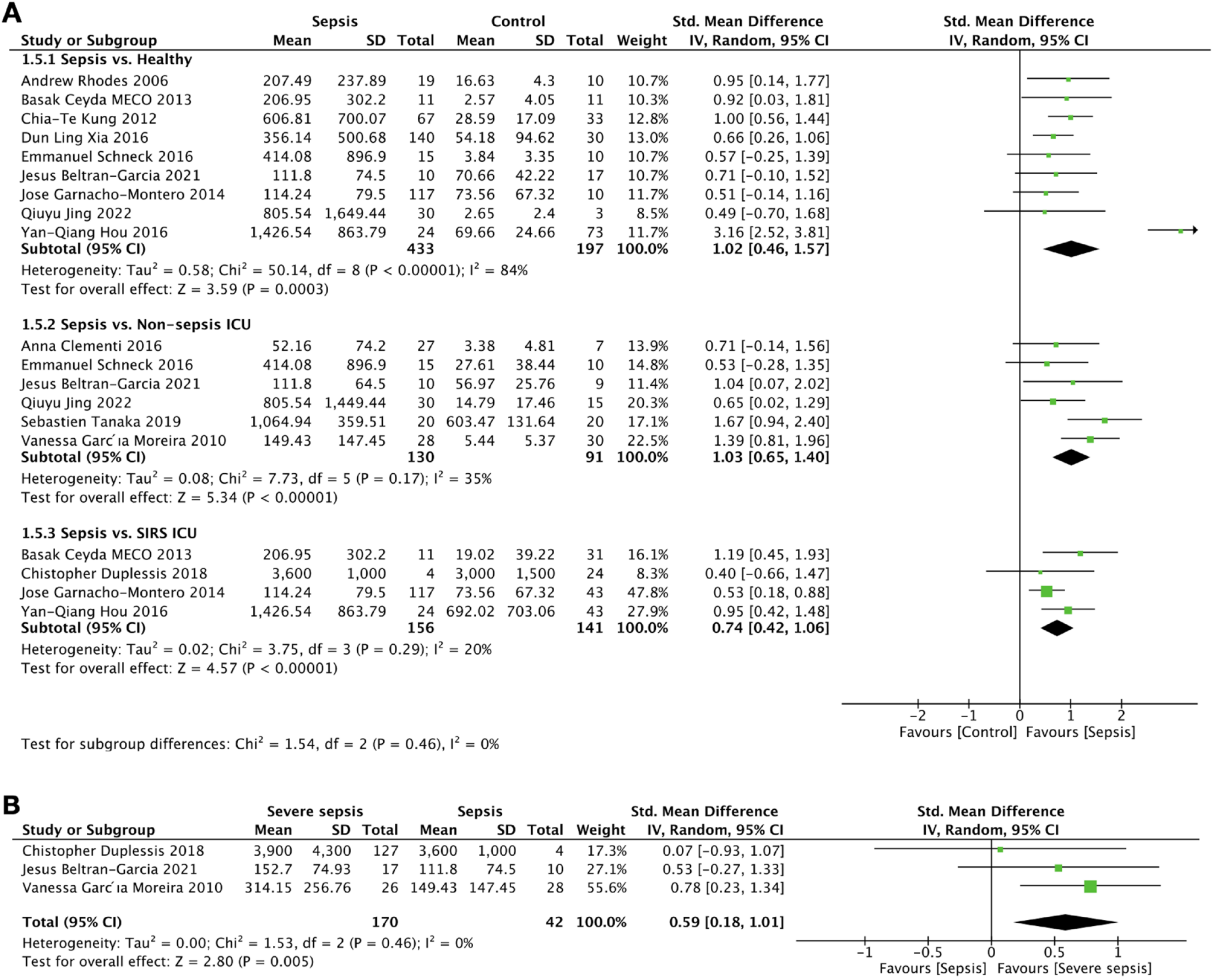
Forest plots of Standardized mean difference (SMD) in cfDNA measurements. (**A**) SMD of cfDNA levels in patients with sepsis compared to controls, (**B**) SMD of cfDNA in severe sepsis compared to uncomplicated sepsis.

A total of 13 studies involving 941 samples were used to differentiate cfDNA concentrations of sepsis versus control. For the comparison, nine and six studies were the comparison between sepsis and the healthy volunteers^21,24,25,28–31,33,37^ and sepsis versus non-sepsis in ICU^22,29,30,33,35,36^, respectively. In parallel, there were four studies that compared patients with sepsis versus non-sepsis with systemic inflammatory response syndrome (SIRS) in ICU^24,26,31,37^. With a comparison to the healthy control, cfDNA levels were significantly higher in patients with sepsis (630 participants) with the standardized mean difference (SMD) = 1.02 (95% CI 0.46–1.57) I^2^ = 84; the mean difference (MD) = 345.15 ng/mL (95%CI 200.86–489.44) and I^2^ = 94% (Fig. 3A).

In comparison with the non sepsis ICU control group, there was also an increase in cfDNA levels in patients with sepsis as evaluated from 221 patients; SMD = 1.03 (95%CI 0.65–1.40) I^2^ = 35%; MD = 163.98 ng/mL (95%CI 75.28–252.68) I^2^ = 87% (Fig. 3A). With the comparison between sepsis versus the ICU SIRS group, there were 297 patients with SMD = 0.74 (95%CI 0.42–1.06) I^2^ = 20%; MD = 255.46 ng/mL (95%CI 1.74–509.18) I^2^ = 79% (Fig. 3A). Among these three studies^26,30,36^, the analysis also exhibited an elevated concentration of cfDNA in patients with severe sepsis when compared between severe sepsis versus sepsis (212 patients, SMD = 0.59 (95% CI 0.18–1.01) I^2^ = 0%; MD = 91.36 ng/mL (95% CI −11.70 to 194.42) I^2^ = 47%) (Fig. 3B).

### Predictive performance of cfDNA for sepsis related mortality

**Figure 4:**
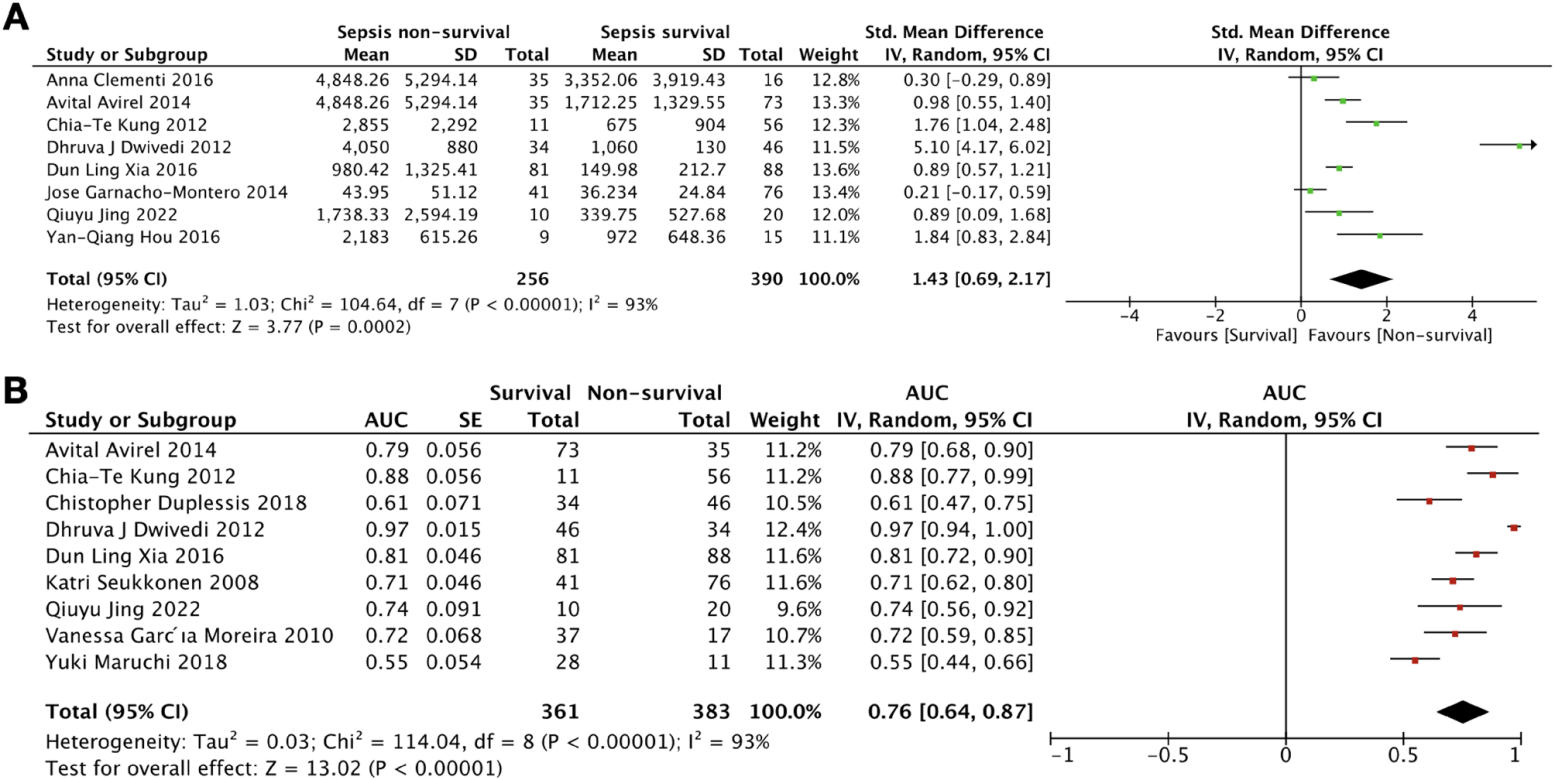
Forest plots of SMD in cfDNA measurements of sepsis survivors compared to non-survivors (**A**) controls and the pooled area under the receiver operating characteristic curve (AUC) for predicting sepsis related mortality (**B**).

The predictive value of cfDNA on sepsis-related mortality was reported by 13 studies involving 936 patients. There were eight studies^22,23,25,28,31,33,37,39^ with a comparison of cfDNA levels between survival versus non-survival groups and nine studies^23,25–28,32,33,36,38^ reporting the area under the receiver operating characteristic curve (AUC) for prediction of mortality. Of these eight studies, there have been reported that sepsis non-survivors demonstrated significantly higher cfDNA levels than sepsis survivors [595 patients; SMD = 1.26 (95% CI 0.08–2.45); MD = 1601.44 ng/mL (95% CI 509.00–2693.88) with significant heterogeneity between the studies (*I*^2^ = 95%)] (Fig. 4A). For prediction of the sepsis-related mortality, the combined AUC of prediction from nine studies was 0.76 (95% CI 0.70–0.86) (Fig. 4B). Subgroup analysis of cfDNA measurement for sepsis-related mortality was also analyzed (Table 2).

**Table 2:** Subgroup analysis of cfDNA measurements of sepsis survivors compared to non-survivors.

	Comparison of cfDNA concentration					AUC for prediction of mortality			
	N	I^2^	cfDNA levels	SMD	95% CI	N	I^2^	AUC	95% CI
All	8	93%	Increase in sepsis non-survival	1.43	0.96–2.17	9	93%	0.76	0.64–0.87
Outcome									
28-day mortality	3	0%	Increase in sepsis non-survival	0.92	0.68–1.16	6	80%	0.64	0.54–0.81
ICU or hospital mortality	5	96%	Increase in sepsis non-survival	1.81	0.31–3.31	4	92%	0.82	0.69–0.95
Time to sample collection									
ICU admission	4	68%	Increase in sepsis non-survival	0.93	0.42–1.44	4	23%	0.80	0.73–0.87
After sepsis	4	97%	Increase in sepsis non-survival	1.98	0.35–3.61	5	96%	0.73	0.54–0.92
Test method									
qPCR	4	83%	Increase in sepsis non-survival	0.75	0.18–1.32	4	56%	0.78	0.70–0.86
Fluorescent	3	24%	Increase in sepsis non-survival	1.10	0.65–1.55	4	72%	0.67	0.55–0.79
Spectroscopy	1	NA	Increase in sepsis non-survival	5.10	4.17–6.02	1	NA	0.97	0.94–1.00

*ICU* intensive care unit, *qPCR* quantitative real-time polymerase chain reaction, *NA* not applicable.

### Diagnostic capacity of cfDNA for the diagnosis of sepsis

**Figure 5:**
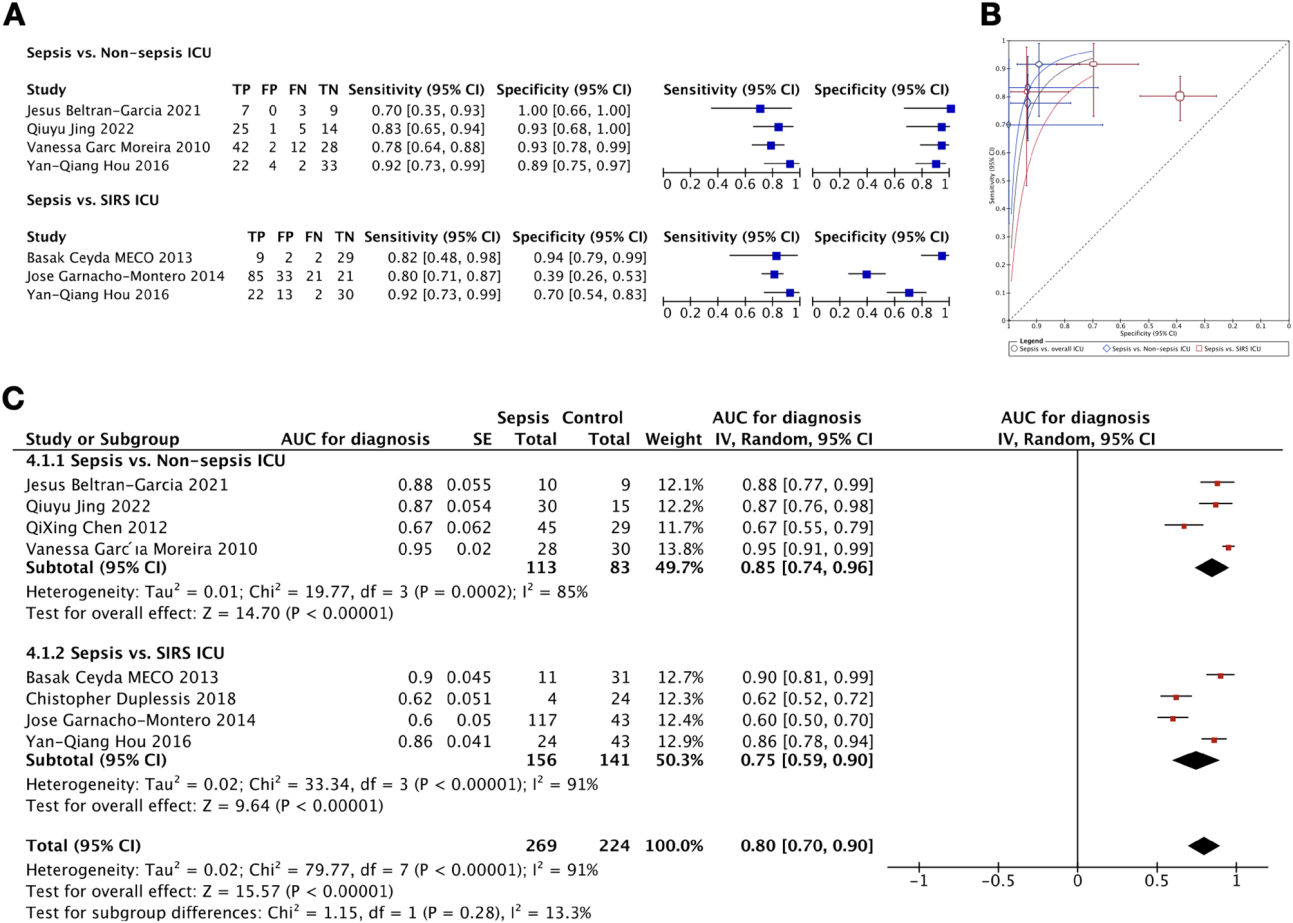
Forest plots of sensitivity and specificity of cfDNA measurements (**A**) and summary receiver operating characteristic curve (SROC; black line = sepsis vs. total ICU control, blue line = sepsis vs. non-sepsis ICU control, red line = sepsis vs. ICU control with SIRS) (**B**) for sepsis diagnosis. Forest plots of the AUC for sepsis diagnosis (**C**).

The diagnostic capacity of cfDNA to distinguish between sepsis versus non-sepsis in ICU was represented in eight studies^24,26,30,31,33,34,36,37^ involving 493 patients. Of these included studies, there was only one study demonstrating the AUC value for sepsis diagnosis^26^. The forest plots of sensitivity and specificity of cfDNA for diagnosis of patients with sepsis and the summary receiver operating characteristic curve (SROC) with pooled diagnostic utility were shown (Fig. 5A, B). The respective values of pooled sensitivity and specificity were 0.81 (95% CI 0.75–0.86; I^2^ = 0%) and 0.72 (95% CI 0.65–0.78; I^2^ = 90.8%), respectively, with pooled calculating diagnostic odd ratio (DOR) at 25.03 (95% CI 5.48–114.43, I^2^ = 80.3%) and the pooled diagnostic accuracy represented by AUC was 0.80 (95% CI 0.70–0.90, I^2^ = 91%) (Fig. 5C).

Additionally, there were studies with subgroup analysis based on the separation into sepsis, non-sepsis, and SIRS in ICU cases. As such, in sepsis versus non-sepsis of ICU cases, the pooled sensitivity and specificity were 0.81 (95% CI 0.73–0.88, I^2^ = 8.9%) and 0.92 (95% CI 0.85–0.97, I^2^ = 0%), respectively, with the pooled DOR and pooled AUC value at 47.45 (95% CI 19.27–116.79, I^2^ = 0%) and 0.85 (95% CI 0.74–0.96; I^2^ = 85%), respectively (Fig. 5A–C). For sepsis versus SIRS of ICU cases, the pooled sensitivity and specificity were 0.82 (95% CI 0.75–0.88, I^2^ = 2.2%) and 0.63 (95% CI 0.54–0.71, I^2^ = 93%), respectively, with the pooled DOR and the pooled AUC at 11.48 (95% CI 1.76–74.89, I^2^ = 83.5%) and 0.75 (95% CI 0.59–0.90; I^2^ = 91%), respectively (Fig. 5A–C).

## Discussion

Sepsis remains an important global health-care problem that still needs better diagnostic and prognostic tests. Although the Surviving Sepsis Campaign acknowledges the possible value of the new biomarkers for sepsis management, the updated guidelines still have no recommendation on the use of any biomarkers for the prognosis prediction or sepsis diagnosis^12^. However, the delay in sepsis management (diagnosis and treatment) increases mortality, prolongs the length of hospital stay, and increases the costs of treatment, highlighting the need for reliable biomarkers for diagnosis of early sepsis and prognosis prediction^13,14^. For cfDNA, our study is the first comprehensive systematic review and meta-analysis which assess the performances of cfDNA as a potential diagnostic and prognostic biomarker for sepsis. Only the longitudinal studies were included to test their quality using QUIPS and QUADAS-2 tools^19^ and our meta-analysis demonstrated the potential of cfDNA for mortality prediction and diagnosis of sepsis. Overall, these results indicate that cfDNA should be further investigated as a measurement to guide clinical evaluations in identifying sepsis and sepsis survival outcomes.

The increased cfDNA in blood during sepsis is possibly released from various types of cell death (apoptosis and necrosis) or cell damage^40,41^, which are a pivotal role in the sepsis pathogenesis^42^. Then, the abundance of cfDNA might be a good indicator for sepsis-induced cell damage that theoretically be correlated with sepsis severity. Indeed, our meta-analysis identified a moderate certainty due to a moderate effect size for the differences in cfDNA levels within 24 h of sepsis. The cfDNA did not only increase in patients with sepsis compared with non-sepsis controls or SIRS (ICU cases), but cfDNA was also elevated in sepsis non-survivors when compared with sepsis survivors. Interestingly, cfDNA levels, measured even at the earliest stages in ICU or at admission (the possible closest time-point to the onset of sepsis), were able to predict mortality rate, as indicated by the pooled AUC for prediction at 0.76 (95%CI 0.64–0.87); an acceptable value for the clinic use^43^. Additionally, patients with initially high cfDNA at admission were also significantly associated with higher mortality rates than the patients with lower cfDNA^27,31^. For the discrimination between sepsis and ICU control with the combined AUC (0.80), pooled sensitivity (0.81), pooled specificity (0.72), and calculating DOR (25.03) indicated cfDNA as a good diagnostic biomarker for sepsis for the practical use^44,45^. However, in subgroup analysis between sepsis versus SIRS, the capacity of cfDNA for sepsis discrimination was decreased as represented by the reduced pooled AUC from 0.80 (sepsis vs. non-sepsis ICU) into 0.75 (sepsis vs. SIRS in ICU), supporting SIRS as an overlapping spectrum of sepsis with significant cell damage^46^. High cfDNA (compared with control) in patients with SIRS, despite an undetectable pathogen, might be an early sign of rapid progression into sepsis after a short follow-up period^22,47^. Similarly, low cfDNA levels in some patients with sepsis might be related to the transition from sepsis to the recovery phase. Considering an indicator of disease severity, cfDNA levels in severe sepsis were higher than the uncomplicated sepsis in a few publications^36^, while other studies could not observe a significant difference^30,36^. Some of this variability may be explained by biological differences between patients and/ or different sensitivity of the used assays among publications^48,49^. For the major difference between real-time qPCR and fluorescent-based assay for measurement, cfDNA was quantified using qPCR for the β-actin housekeeping gene, thus detecting a subset of cfDNA in nuclear DNA, but not mitochondrial DNA (mt-DNA) or microbial DNA. Meanwhile, the fluorescent-based assay detects all of cfDNA (nuclear DNA, mt-DNA, and microbial DNA)^49^ showing a high certainly due to a low effect size (I^2^ = 24%) for differences in cfDNA levels to detect severe subjects and demonstrating the highest SMD (subgroup analysis in Table 2). Although the tests for cfDNA are relatively simple and inexpensive, especially in fluorescent-based assay, the cfDNA measurement in routine clinical practice is still uncommon. The combination of cfDNA with the current sepsis scoring system may yield even stronger predictive power. Indeed, the current clinical scoring of sepsis, such as Acute Physiology and Chronic Health Evaluation (APACHE) II and Sequential Organ Failure Assessment (SOFA), exhibit only a moderate discriminative power for sepsis mortality prediction with that AUC at 0.6 to 0.7^50,51^.

The cfDNA in sepsis might be produced from cell damage from inadequate oxygenation (hypoxia) and cell death^52–54^ in both parenchymal and immune cells, especially granulocytes and cells from several organs, as determined by the cfDNA methylation profile^55^, that might be different from patients with trauma^56^. While the sepsis-associated cfDNA is possibly primarily released by activated neutrophils due to the prominent production of polymorphonuclear cells (PMN) in response to pathogens, trauma-associated cfDNA mostly originate from the injured cells because of the less PMN responses against the damaged host molecules^56^. More profound neutrophil extracellular traps (NETs), the extracellular decondensed chromatin with antimicrobial molecules, in early sepsis compared with the trauma-induced SIRS^57^ might be one of the main sources of sepsis-associated cfDNA^58^, supporting NETs dysregulation in several diseases^57–59^. Also, NETs are one of the mechanisms responsible for acute respiratory distress syndrome (ARDS), vascular damage, and microthrombi leading to multiorgan failure and death^59^ that are common in sepsis. Indeed, the roles of cfDNA as a major crosslink between inflammation and coagulation, referred to as “immunothrombosis”^60,61^, partly through the induction of Toll like receptor-9 (TLR-9)^62,63^, and coagulation system by activation of primary and secondary hemostasis^64,65^ are well-known. In animal studies, cfDNA-mediated severe sepsis is demonstrated as scavengers of cfDNA attenuate cfDNA-induced inflammation^39^ and sepsis severity^66^ supporting cfDNA as a key contribution in sepsis and the use of cfDNA as the biomarker or therapeutic target. Indeed, compared to other potential biomarkers for sepsis diagnosis such as procalcitonin (PCT), our findings exhibited that calculating the DOR of cfDNA was higher than PCT showed calculating DOR of 12.5 from meta-analysis, whereas pooled sensitivity and specificity were similar^67,68^. However, there are preanalytical considerations in sample collecting for cfDNA analyses as preservation and process of preparation might interfere with cfDNA levels^49^. Additionally, the kinetic of cfDNA in sepsis is still uncertain. The fetal cfDNA is rapidly cleared from maternal plasma after delivery^69^ and increased cfDNA from hemodialysis returns to baseline within 30 min after stopping the session^70^, suggesting that cfDNA is eliminated through the reticuloendothelial system in the livers and spleens or clearance through glomerular infiltration in the kidneys^69^. Because alteration in blood levels of biomarkers with a long half-life is too slow to represent a real-time patient’s situation^12^, the short half-life of cfDNA together with the capacity to induce inflammation results in the use of cfDNA as an indicator of cell damage in several conditions with immediate emergencies^71^. In our systemic review, the included studies^29,31,35,39^ demonstrated that cfDNA measurement in sepsis within 24 h of admission was the highest value observed and also showed the highest property of mortality prediction. More studies on this topic are warranted.

Nevertheless, several limitations of the current study should be discussed. First, there are risks of bias regarding patient misclassification, partly due to variations in the reference standard and sepsis criteria in the included studies such as severe sepsis defined in the Sepsis-1 and 2, but not classified in the Sepsis-3. Some studies included patients who were clinically diagnosed with sepsis without microbiological evidence^12^. Second, there was a combination of the studies with different cfDNA measurement assays that could not suggest the difference among these assays. Third, our study was unable to conduct the ideal cut-off point of circulating cfDNA levels due to the limited of reporting on the raw data to map out the ROC curve. Fourth, there was no comparison between cfDNA and other sepsis biomarkers. Finally, only studies published in English were included. Despite these limitations, cfDNA was demonstrated as a good indicator for sepsis diagnosis and mortality prediction. Then, we proposed cfDNA as a part of an interesting multi-biomarker panel for early sepsis diagnosis and sepsis outcomes. Future studies of cfDNA in sepsis are interesting.

## Conclusions

The comprehensive meta-analysis includes current studies to demonstrate the use of cfDNA levels as a biomarker in critically ill conditions of sepsis. In this study, cfDNA concentrations were significantly higher in sepsis than the control with the presentation of prognostic and diagnostic capacities. We encourage further studies in this area with the aforementioned guidelines together with the use of cfDNA.

## Data Availability

The datasets used and/or analyzed during the current study are available from the corresponding author on reasonable request.
